# Supplementary material for: PyuARF16/33 Are Involved in the Regulation of Lignin Synthesis and Rapid Growth in Populus yunnanensis
Source: Genes (Basel). 2023 Jan 21;14(2):278. doi: 10.3390/genes14020278 (PMC9956056; doi:10.3390/genes14020278)

Table S1 The gene-specific primers for RT-qPCR used in this study

**Gene name**	**Forward Primer(5' to 3')**	**Reverse Primer(5' to 3')**
PyunARF33	GATGCAGCAGAGGCTAGATCC	AGCTGCAACAGTGGATTGGG
PyunARF5	TGCAGCAGCGCATTCAGTAT	CGCCTGCAAAGATGGAGAGT
PyunARF27	GGGAACCTCGTGACAAGCC	CAGCTGCTCTACTTGGCCC
PyunARF35	TTGTCAGAAATGGCTGGTCAC	AGCTAAAGAGTGTCACGGCA
PyunARF18	CCAGCAGCAGCAGAATCAAC	CTGTCCAAGTTGACCAGCCT
PyunARF21	GGAGTCCGCAGATACATGGG	CCTGGCTGCTTAGGGAACTT
PyunARF3	GAGCTCCTCAATGCATGGTG	GTTGATCAGTGCCACCATCC
PyunARF4	TGCAACCACCATCTCAGGAGC	GCCTCACACCCACCATCAAG T
PyunARF16	TCGAGGACAACCCAAGAGG	AGGCATGAAAGTTTGAAGAC G
PyunARF13	TTCTCTGTTCCTCGTCGTGC	ACAAACACACTCCAACCCGT
PyunARF17	CAATGCAACCGCCAACTCAG	GCCTCACACCCACCATTAAC T
PyunARF34	TATGGTGGTACCGCACAAGA	TGATTCTGGTTGGTCCAGGC
HIS	TTTAAGACTGATCTGCGTTTCC	GAACAGCCCAACAAGGTATG

Table S2 The proportion of Gln(Q),Ser(S),Leu(L),Pro(P) and Thr(T) in MR domain of *PyuARFs* gene. The proportion of Gln (Q), Ser (S), Leu (L), Pro (P), Thr (T) determines whether the MR Domain of ARFs has transcriptional activation or inhibition.

>PyuARF17/207-279

NR SPFT I FY N PRAD FV I P LI K FR KA VFGQV SVGMRF G M M FETE E S GKRRY M GTI V G IS DDP L RWPGS K WR N LQ

Gln (Q) 2 2.7%
Ser (S) 5 6.8%
Leu (L) 3 4.1%
Pro (P) 5 6.8%
Thr (T) 3 4.1%

>PyuARF4/207-279

NRSSFTIFYNPRADFVIPLIKFRKTVFGQVSVGMRFGMMFETEESAK RRY M GTI V G NS DDP L RWPGS K WR N LQ

Gln (Q) 2 2.7%
Ser (S) 6 8.2%
Leu (L) 3 4.1%
Pro (P) 4 5.5%
Thr (T) 4 5.5%

>PyuARF21/207-279

NNSPFTIFYNPRAEFVIPFSKYNKALYTQVSLGMRFRMMFETEESGV RRY M GTI T G IS DDPVRW KN S Q WR N LQ

Gln (Q) 3 4.1%
Ser (S) 6 8.2%
Leu (L) 3 4.1%
Pro (P) 4 5.5%
Thr (T) 5 6.8%
>PyuARF34/207-279
NN SPFT I FY N PSA EFV I P FS K YN KAM YTQG S L GMRF R M M F T TE E S GVRRY M GTI T G IS DDPVRW KN S Q WR N LQ
Gln (Q) 3 4.1%
Ser (S) 7 9.6%
Leu (L) 2 2.7%
Pro (P) 4 5.5%
Thr (T) 6 8.2%
>PyuARF18/207-279
NN SPFTVFY N PRAEFV I P LA K YY KA VYSQI S L GMRF R M M FETE E S GT RRY M GTI T G IS DDP A RW KN S Q WR N LQ
Gln (Q) 3 4.1%
Ser (S) 6 8.2%
Leu (L) 3 4.1%
Pro (P) 4 5.5%
Thr (T) 5 6.8%
>PyuARF35/207-279
NNSPFTVYYNPRAEFVIPLAKYYKAVYSQISLGMRFRMMFETEESGTRR HM GTI T G IS DD A VRW KN S Q WR N LQ
Gln (Q) 3 4.1%
Ser (S) 6 8.2%
Leu (L) 3 4.1%
Pro (P) 3 4.1%
Thr (T) 5 6.8%
>PyuARF13/165-235
PAQELIARDLHDVEWKFRHIFRGQPKRHLLTTGWSVFVSAKRLVAGDSV LFIWNEKNQLLLGIRRATRPQT
Gln (Q) 4 5.6%
Ser (S) 3 4.2%
Leu (L) 9 12.7%
Pro (P) 3 4.2%
Thr (T) 4 5.6%
>PyuARF33/207-279
T N S C FTVFY N PRAEFV I P LS K YV KA VFHRI SVGMRF R M L FETE E SS VRRY M GTI T G TS DDPVRWP N S H WRS VK
Gln (Q) 0 0.0%
Ser (S) 8 11.0%
Leu (L) 2 2.7%
Pro (P) 4 5.5%

Thr (T) 6 8.2%

>PyuARF3/207-279

T N S R FT I FY N PRAEFV I P LA K Y LKA VYYRV SVGMRF R M M FETE E SS VRRY M GTI T G IS DD V VRWP N S Q WRS VK

Gln (Q) 1 1.4%

Ser (S) 7 9.6%

Leu (L) 2 2.7%

Pro (P) 3 4.1%

Thr (T) 5 6.8%

>PyuARF27/207-279

T N S R FT I FY N PRAEFV I P LA K Y LKA VYYRV SVGMRF R M L FETE E SS VRRY M GTI T G IS DD VA RWP N S L WRS VK

Gln (Q) 0 0.0%

Ser (S) 7 9.6%

Leu (L) 4 5.5%

Pro (P) 3 4.1%

Thr (T) 5 6.8%

>PyuARF16/207-279

T N S R FT I FY N PRAEFV I P LV K YI KA VYHRV SVGMRF R M L FETE E SS VRRY M GTI T G IS DDPVRWP N S H WRS VK

Gln (Q) 0 0.0%

Ser (S) 7 9.6%

Leu (L) 2 2.7%

Pro (P) 4 5.5%

Thr (T) 5 6.8%

>PyuARF5/207-279

T N S R FT I FY N PR TEFV I P LV K YI KA VYHRV SVGMRF R M L FETE E SS VRRY M GTI T G IS DDP A RWP N S H WRS VK

Gln (Q) 0 0.0%

Ser (S) 7 9.6%

Leu (L) 2 2.7%

Pro (P) 4 5.5%

Thr (T) 6 8.2%

Figure S1 Sequence alignment and corresponding domain of *PyuARFs*. ARFs has three main domains: B3, MR And AUX/IAA

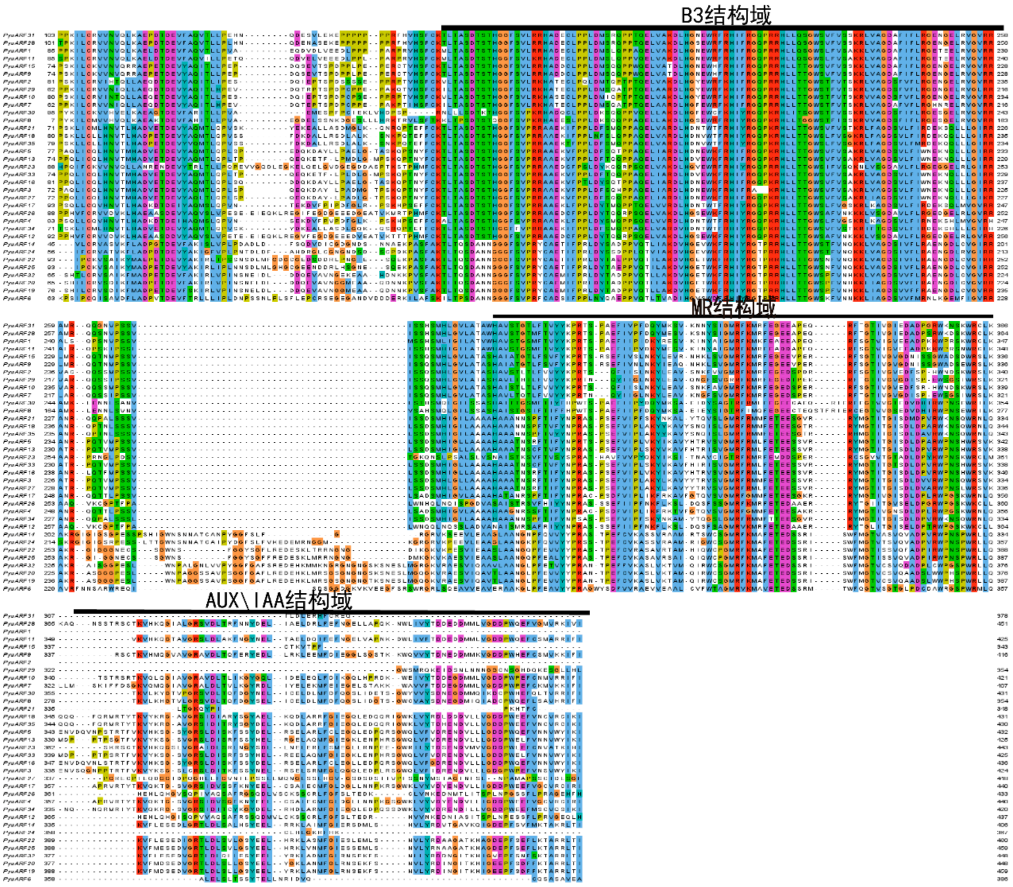

Figure S2 The number and types of Motifs of *PyuARFs.*

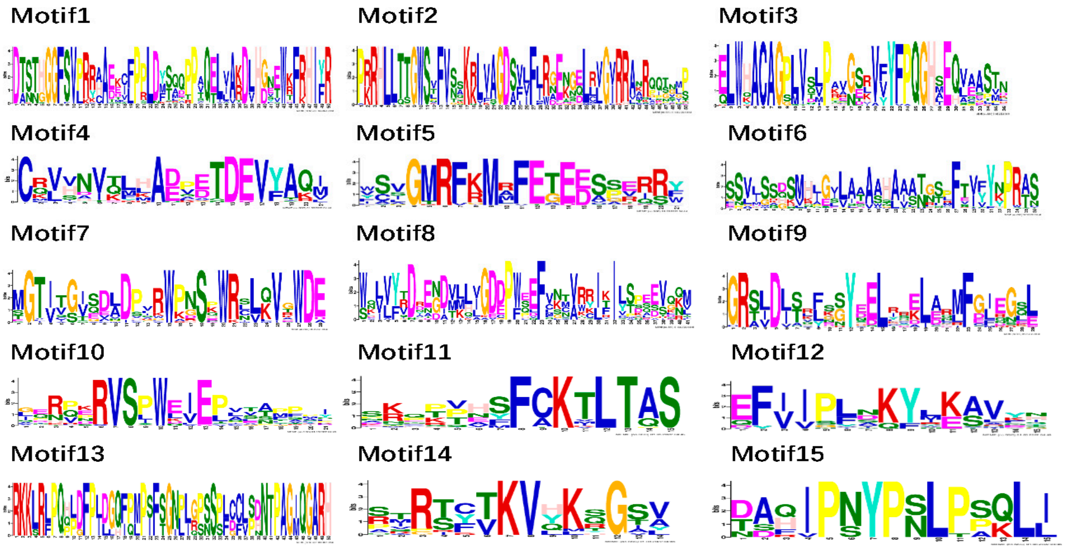

Supplement: Supplementary file 1 [file genes-14-00278-s001.zip › genes-2123343-supplementary.pdf]
